# A Rare Case of Myxedema Coma Precipitated by a Single Dose of Haloperidol in a Patient With Paranoid Schizophrenia

**DOI:** 10.7759/cureus.45190

**Published:** 2023-09-13

**Authors:** Manuel Menendez, Temilola Majekodunmi, Farah Chohan, Aaron Perez-Castaneda, Hiram Maldonado Rivera, Eugenio Angueira-Serrano, George Michel

**Affiliations:** 1 Internal Medicine, Ross University School of Medicine, Bridgetown, BRB; 2 Internal Medicine, Larkin Community Hospital, South Miami, USA; 3 Endocrinology, Larkin Community Hospital, South Miami, USA; 4 Endocrinology, Larkin Community Hospital Palm Springs Campus, Hialeah, USA

**Keywords:** thyroid replacement therapy, endocrine disorder, severe hypothyroid states, antipsychotic use, haldol, thyroid dysfunction, hypothalamic-pituitary axis, hypothyroid myxedema coma

## Abstract

Myxedema coma is a rare and potentially life-threatening condition that occurs when severe hypothyroidism is untreated or inadequately managed. It is characterized by a rapid drop in mental status, hypothermia, respiratory failure, hypotension, and other symptoms of severe metabolic dysfunction. The condition primarily affects older women with a history of thyroid dysfunction but can occur in any age or gender group. A common trigger for this condition is seen with a drop in atmospheric temperatures or during the cold winter months. However, a rare, poorly documented trigger is recent antipsychotic use in severe hypothyroid states. The diagnosis of myxedema coma requires prompt recognition and treatment, as delays can result in significant morbidity and mortality which is the objective of this case report.

## Introduction

Myxedema coma is a medical emergency resulting from severe hypothyroidism, which can be life-threatening if left untreated. Hypothyroidism is a condition in which the thyroid gland fails to produce enough hormones, leading to a range of symptoms such as fatigue, weight gain, and cold intolerance. Myxedema coma occurs when hypothyroidism reaches the severity level of causing a significant decline in mental and physical function, leading to coma or even death. The condition primarily affects older women with a history of thyroid dysfunction but can occur in any age or gender group. A common trigger for this condition is seen with a drop in atmospheric temperatures or during the cold winter months [1]. The diagnosis of myxedema coma can be challenging, as its symptoms can mimic other medical emergencies such as sepsis or stroke in a clinical setting. One poor prognostic factor for patients in a myxedema coma is seen with hypothermia [2]. Treatment involves the immediate transfer to the ICU, replacement of thyroid hormones, supportive care [1], and managing any precipitating factors such as infection or medication misuse. Such misuse of medications includes the use of sedatives, as well as antipsychotics, as seen in our case with the use of haloperidol [3]. Haloperidol is a medication that is a primary treatment option for patients with schizophrenia. It is also known as a first-generation antipsychotic that balances the levels of dopamine in the brain to improve thinking, mood, and behavior. This, however, when used in a severe hypothyroid state, can precipitate a myxedema coma [4]. Despite its rarity, myxedema coma is a critical condition that requires heightened awareness and immediate intervention to optimize patient outcomes.

## Case presentation

Our patient is a 78-year-old Hispanic female with a past medical history of dementia, diabetes mellitus, hypothyroidism, hypertension, and paranoid schizophrenia who was brought to the emergency department (ED) in April by ambulance from her assisted living facility (ALF) for right lower extremity pain in the afternoon. The patient's list of home medications included: allopurinol 100 mg daily, acetylsalicylic acid (ASA) 81 mg daily, furosemide 20 mg PO daily, losartan potassium 50 mg daily, metformin 850 mg BID, metoprolol tartrate 50 mg BID, mirtazapine 30 mg HS, and risperidone 0.25 mg HS. On review of systems, the patient endorsed fatigue, malaise, swelling of the right leg, and palpitations but denied any chest pain or shortness of breath. At the time of the initial examination, the patient was awake, alert, and oriented to person and place (her baseline). Vitals at the time of admission were as follows: temp 97.5, pulse 75, oxygen saturation 97% on room air, respiratory rate 15, BP 152/62. On examination, the right lower limb was swollen and tender, warranting a venous Doppler ultrasound which revealed a partially occlusive age-indeterminate deep vein thrombosis (DVT) in the right popliteal vein. Other labs ordered showed thyroid-stimulating hormone (TSH) 149 mlU/L, free thyroxine (T4) 0.34 ug/dL, glycosylated hemoglobin (HbA1c) 9.8%, brain natriuretic peptide (BNP) 542 pg/mL, and hemoglobin (Hgb) 9.8 g/dL (Table 1).

**Table 1:** Pertinent labs on admission TSH: thyroid-stimulating hormone; T4: thyroxine; Hgb: hemoglobin; HbA1c: glycosylated hemoglobin; BNP: brain natriuretic peptide

Lab values	On admission
TSH	149 mlU/L
Free T4	0.34 ug/dL
Hgb	9.8 g/dL
HbA1c	9.8%
BNP	542 pg/mL

The patient was started on enoxaparin 120 mg subcutaneously (SQ) every 12 hours for DVT treatment, dexamethasone 4 mg intravenous push (IVP), and levothyroxine 200 mcg IVP to manage her severe hypothyroidism, and was admitted to the medical floor. A random cortisol level was ordered, which resulted in 14.6, essentially ruling out adrenal insufficiency. Endocrinology was consulted for input on optimizing patient care.

Following admission, she became increasingly agitated and tried to pull her IV lines several times. Consequently, haloperidol 5 mg intramuscularly (IM) was administered to calm her down. Notably, haloperidol was administered prior to dexamethasone and levothyroxine. The following morning (day #2), a rapid response code was called as the patient was noted to be somnolent cold to the touch, with a core body temperature of 95°F and heart rate (HR) of 52. The patient was promptly stabilized and transferred to the intensive care unit (ICU) for close observation and medical management. Arterial blood gas (ABG) analysis at the time of admission to ICU were as follows: pH 7.36, pC02 56, P02 105, BE(B) 3.9, HCO3 31. A Myxedema Coma Screening Tool Score was calculated to be 60 (10 points for temperature 32-35, 15 points for obtunded state, 10 points for bradycardia 50-59, 5 points for constipation, 10 points for precipitating event, 10 points for hypercarbia). A score of more than or equal to 60 is suggestive/diagnostic of myxedema coma, a score of 25-59 indicates a high risk for myxedema coma, and with a score less than 25 myxedema, coma is unlikely. Her antipsychotic, sedatives, and antihypertensive home medications were all suspended; oral antiglycemics were changed to sliding scale and basal-bolus insulin. Dexamethasone 4 mg IVP was administered followed by levothyroxine 300 mcg IVP. The patient was kept warm using warming blankets (temperature 97.9°F). A repeat, free T4 after the commencement of treatment was 0.72 ug/dL; however, the patient remained awake, alert, and oriented x1 (AAOx1; only to self).

On day #3, the patient continued to show improvement clinically, in free T4 levels (0.34 on day 1, 0.72 on day 2, 0.88 on day 3) and was safely downgraded to the med-surg floor. Though more oriented, the patient displayed intermittent aggression, warranting a psychiatric consult. After a thorough evaluation by both teams, a decision was made to start the patient on quetiapine 25 mg PO BID. During hospitalization, her DVT management with enoxaparin was switched to Eliquis (loading dose of 10 mg BID); however, a hematoma was noted on the right arm after which the Eliquis was suspended and the patient was started on heparin 5000u q12h. Per the interventional radiology team, no surgical intervention was warranted at this time. Imaging was ordered including a soft tissue ultrasound of the right upper extremity that showed a medial forearm mass which appeared heterogenous, measuring approximately 4.24 x 3.12 cm. A venous ultrasound of the right arm was also performed that showed no evidence of DVT, with mild right arm fluid infiltration. Due to improvement, the Eliquis was resumed (dose 10 mg PO BID).

By day #4, free T4 was 1.09 ug/dL. Levothyroxine 100 mcg IVP was administered and the patient was transitioned to levothyroxine 200 mcg PO Q daily. Dexamethasone was also decreased from 4 mg IVP to 2 mg IVP, eventually to be tapered off over the next 10 days. With continued clinical and lab improvements noted on day #5, the patient was safely transitioned to PO medications, repeat-free T4 was 1.38, and a plan was made to discharge her back to the assisted living facility with a return to baseline function. Her home medications metformin (850 mg BID), losartan potassium (50 mg daily), and allopurinol (100 mg daily) were continued at the same dose. Metoprolol tartrate was changed from 50 mg BID to 25 mg BID, and furosemide and aspirin were discontinued. In light of her elevated A1C level (9.8), she was also started on sitagliptin 100 mg PO daily and empagliflozin 10 mg PO daily with an A1C goal of 7.5-8.0% in order to avoid the risk of hypoglycemia. Levothyroxine 200 mcg PO daily was also started. The loading dose of Eliquis 10 mg PO BID was to be transitioned to a maintenance dose at 5 mg BID after 7 days, and prednisone to be tapered over the next 10 days. Her antipsychotics were changed from risperidone and mirtazapine to quetiapine fumarate 25 mg PO at bedtime (low dose as recommended by the psychiatry team). The patient was instructed to follow up with her primary care physician, psychiatrist, and endocrinologist in 7-10 days following discharge.

## Discussion

Myxedema coma is an endocrine emergency requiring prompt diagnosis, identification of underlying factors, and treatment of the severe hypothyroid state [5]. While we know that myxedema coma is a rare but life-threatening complication of severe hypothyroidism that may occur due to various causes (including thyroid gland dysfunction, iodine deficiency, autoimmune disorders, or pituitary or hypothalamic dysfunction), triggers include infection, trauma, hypothermia, and drugs that interact with the thyroid hormones or its function (e.g. anabolic steroids, tyrosine kinase inhibitors, oral contraceptive pills, amiodarone, dopamine agonists, etc.). Presenting symptoms typically include lethargy, confusion, hypothermia, bradycardia, and respiratory depression; all of which require urgent medical intervention, with hormone replacement therapy [6].

According to the literature [5-7], myxedema coma is more prevalent in females with a history of hypothyroidism, with a general prevalence of about 1% of the hypothyroid population [7]. Postulated pathophysiology includes reduced respiratory drive secondary to the hypothyroid state which leads to increased CO2 retention; an inappropriate release of antidiuretic hormone (ADH) leading to myxedema; and overall hypothermia from the excessively reduced metabolic rate leading to hypothermia. Mortality rates of myxedema coma range between 20-50% [5,7].

On presentation, our patient was obviously undergoing a DVT event; hence, her workup and management were geared towards that diagnosis. Notwithstanding, and rightfully so, further clinical evaluation revealed that although the patient had a history of hypothyroidism and paranoid schizophrenia, these were both undermanaged. It was interesting to note that although the patient admitted to a few symptoms of hypothyroidism and the initial TSH and T4 gave that away, she did not seem to be in a life-threatening situation. This may suggest that hypothyroid patients may remain within an unknown tolerance range provided the threshold is not exceeded. There are no known clinical levels for this threshold to date. Hence, the only way to avoid a patient tipping over into the fatal spectrum is proper management. However, there have been a few cases of patients with asymptomatic hypothyroidism going into myxedema coma [5]. Our case presents a rare but possible trigger for myxedema coma in the geriatric population, medication-induced myxedema coma. With the advances in medicine in recent decades, clinicians are seeing an increased prevalence of once-fatal conditions, and hence, a higher life expectancy for patients with chronic diseases. With this grows the need for advancements in knowledge and awareness of medication side effects and/or polypharmacy, with the ever-increasing complexity of patient care.

Although myxedema coma is not commonly associated with psychiatric medication use, there have been some reports of the condition occurring in patients with hypothyroidism secondary to the use of antipsychotic medications, such as haloperidol, which is used to treat various psychiatric disorders, including schizophrenia and bipolar disorder [8]. The most commonly used antipsychotic in hospitals today (particularly in the ED) is haloperidol, =due to the ease of administration and quick onset of action. However, it carries a black box warning of increased risk of death for patients with dementia-related psychosis. Jerry et al. reported a case of a 58-year-old schizophrenic patient with previously undiagnosed hypothyroidism who presented with symptoms of fatigue and mental decline that continued to worsen [9]. Initially, his symptoms were attributed to an increased dose of tranquilizer and mood stabilizer started about a week prior to treating his poorly controlled psychiatric symptoms. The patient soon became unresponsive and lab work was suggestive of severe hypothyroidism, with the clinical symptoms pointing towards myxedema coma. Despite resuscitative measures, the patient’s clinical condition continued to deteriorate resulting in his death. A retrospective study conducted in Asia with about 188 patients suffering from bipolar disorder showed that a combination of antipsychotics and psychotropics led to a significant drop in free T4, compared to the use of antipsychotics alone [8]. The study also postulated that psychotropic drugs suppress the hypothalamic-pituitary-thyroid (HPT) axis, as well as the hypothalamic-pituitary-adrenal (HPA) axis function. However, the study was limited in that it did not evaluate the effects of antipsychotics alone on the function of the HPA and HPT axis. More study is needed to evaluate the extent to which antipsychotics inhibit the release of hypothalamic and hypopituitary hormones in relation to thyroid hormone levels. In our case, thanks to the input from the psychiatry consult, our patient was safely managed with quetiapine, which is one of the safest medications to give in hypothyroid patients, without triggering further hypothyroidic events in our patient.

Interestingly, an early publication on myxedema coma in 1969 [7], proposed the use of triiodothyronine (T3) or T4 together with high-dose corticosteroids in treating myxedema coma. Not much has changed in the treatment modality since then, apart from the preferred administration of T4 in the USA over T3. The Myxedema Coma Screening Tool Score in our case was calculated to be 60 (10 points for temperature 32-35, 15 points for obtunded state, 10 points for bradycardia 50-59, 5 points for constipation, 10 points for precipitating event, 10 points for hypercarbia). A score of more than or equal to 60 is suggestive/diagnostic of myxedema coma; a score of 25-59 indicates a high risk for myxedema coma, and with a score less than 25 myxedema coma is unlikely [10]. Our patient was treated with a high-dose corticosteroid and T4 with a good outcome. Her psychiatric comorbidity responded well to quetiapine (a second-generation antipsychotic with less dopaminergic antagonistic effect) [11], and within 1 week, the patient was back to her baseline, and stable enough for discharge. Here we can tell that due to her diagnosis of drug-induced myxedema coma, due diligence was taken in prescribing a more suitable antipsychotic with a lower probability of side-effects. Routine labs were ordered to closely monitor improvement in TSH and T4 levels while clinically assessing the patient for clinical improvements daily.

Although our patient was briefly managed in the ICU, she was promptly treated and de-escalation to the floor occurred within 72 hours. It is worth noting that the length of ICU stays could greatly impact patient outcomes. Studies have shown that in critically ill patients requiring ICU care, post-ICU morbidity, including depression and long-term cognitive changes tends to increase [12]. Hence, it is imperative to quickly identify the diagnosis, remove offenders, and commence treatment immediately to reduce ICU stays and improve overall post-ICU quality of life for patients.

## Conclusions

This case report presents a rare instance of myxedema coma triggered by medication use in a geriatric patient, emphasizing the importance of considering this less common etiology in hypothyroid patients on specific medications. The report offers a comprehensive account of the patient's clinical course, highlighting successful interdisciplinary collaboration in her management and the value of prompt diagnosis and treatment to minimize ICU stays and post-ICU morbidity. While the report provides practical insights into treatment strategies and the use of a clinical scoring tool, its single-case nature limits generalizability, and further research on medication-induced myxedema coma mechanisms and risk factors may be warranted to better understand this unusual condition.
